# The Effectiveness of Bivalent COVID-19 Vaccination: A Preliminary Report

**DOI:** 10.3390/life13102094

**Published:** 2023-10-21

**Authors:** Ssu-Yu Chen, Chien-Yu Lin, Hsin Chi, Shun-Long Weng, Sung-Tse Li, Yu-Lin Tai, Ya-Ning Huang, Hsiang Huang, Chao-Hsu Lin, Nan-Chang Chiu

**Affiliations:** 1 Hsinchu MacKay Memorial Hospital, Hsinchu City 300, Taiwan; 2 Hsinchu Municipal MacKay Children’s Hospital, Hsinchu City 300, Taiwan; 3 Department of Medicine, MacKay Medical College, New Taipei City 251, Taiwan; 4 MacKay Children’s Hospital, Taipei 104, Taiwan

**Keywords:** COVID-19, SARS-CoV-2, vaccination, bivalent, updated vaccine, BA.1, BA.5

## Abstract

Vaccination has been a game-changer in the long battle against COVID-19. However, waning vaccine-induced immunity and the immune evasion of emerging variants create challenges. The rapid-fire development of bivalent vaccines (BVs), comprising ancestral strains and a new variant, was authorized to prevent COVID-19, but the effectiveness of the updated vaccines remains largely unclear. Electronic databases were searched to investigate the immunogenicity and reactogenicity of BVs in humans. As of March 2023, 20 trials were identified. Compared with monovalent vaccination, the induced immunogenicity against ancestral strains was similar. The BVs demonstrated approximately 33–50% higher immunogenicity values against additional variant strains. An observational cohort study showed the additional clinical effectiveness of the BVs. The adverse events were similar. In conclusion, our systematic review found that the BVs had equal immunogenicity against ancestral strains without safety concerns. Approximately 33–50% increased additional antibody titers and clinical effectiveness against additional variant strains were observed in subjects with a BV vaccine with moderate heterogeneity, especially for BA.1-containing BVs.

## 1. Introduction

The coronavirus disease 2019 (COVID-19) pandemic has existed for over three years. As of May 2023, it has created substantial impacts in all aspects, and more than 683 million residents were infected, with estimates of up to 20 million deaths [1,2]. The virus was highly contagious, and the case fatality rate was approximately 2% for the ancestral strain. Various strategies and measurements were implemented to control, contain, and mitigate the pandemic, including lockdowns, social distancing, non-pharmaceutical interventions, antiviral agents, immune modulators, and vaccines [3,4,5]. The mainstay measurements varied in different areas with different epidemics, resources, and times.

Vaccination is believed to be a game-changer, and several COVID-19 vaccines with different platforms were developed and received emergency use authorization since 2021, including a protein-based vaccine, an inactivated vaccine, an adenovirus-vectored vaccine, and an mRNA vaccine [6,7,8]. The initial COVID-19 vaccines were effective and contributed to reductions in symptomatic visits to the emergency department, hospitalization, ventilator use, and mortalities [4,9,10]. The clinical efficacy was up to 95% in a phase 2 study of mRNA vaccines [11,12]. Highly effective vaccination with broad coverage is crucial for easing socially restrictive measurements and non-pharmaceutical interventions, and people are eager to return to normal life. However, a rapid waning of vaccine-induced immunity after several months has been observed, and a booster dose is required to maintain adequate immunity [13]. Moreover, virus mutation is an important concern, and rapid viral mutations occur. The replacement of the predominant viral mutant has received global attention, and immune evasion is evident for Omicron lineages [14]. A moderate mutation of the spike protein results in immune evasion and inadequate vaccine protection [13]. In brief, protecting immunity induced by the primary series with the original COVID-19 vaccine is unsatisfactory during the Omicron pandemic, and a booster should be considered.

We are working hard to develop a new bivalent COVID-19 vaccine to overcome this situation. The updated bivalent vaccine (BV) comprises two antigens of different virus variants to provide broader protection and more specific immunogenicity for newly mutating variants [15]. Different components of updated bivalent COVID-19 vaccines have been investigated, including ancestral, D614G, Alpha, Beta, Omicron sub-lineage BA.1, and BA.4/BA.5 variants [15,16,17]. The mutating Omicron variant spread rapidly to all countries, and the emergency use authorization of the BV against Omicron variants was accelerated. An mRNA vaccine comprising ancestral and BA.4/BA.5 strains was approved in the United States on 31 August 2022. However, the approval of a new bivalent vaccine against BA.4 and BA.5 was authorized based on the findings of animal studies of BA.4/BA.5 vaccines and human studies of BA.1-containing vaccines. There are no available human data from before the authorization, and there are concerns about the safety and real-world effectiveness of the updated BA.4/BA.5 vaccine. Some human studies were conducted after authorization, and we conducted this systematic review to investigate the safety and effectiveness of the updated bivalent vaccine.

## 2. Materials and Methods

### 2.1. Study Design and Literature Search

We conducted this study in accordance with the Preferred Reporting Items for Systematic Reviews and Meta-analyses (PRISMA) guideline, and written informed consent was waived for a systematic review without identifiable patient information [3,18,19]. The “PICO” of the present study comprised people with COVID-19 vaccination; intervention with a bivalent vaccination, including bivalent COVID-19 vaccines with all kinds of platforms and components, a comparison of people with an original monovalent vaccination or a lack of vaccination; and the outcomes were safety, immunogenicity, and clinical effectiveness. As of March 2023, we have searched electronic medical databases, including PubMed/Medline, Embase, and the Cochrane Library, and preprint medical databases such as medRxiv. We used comprehensive keywords, such as “COVID-19”, “COVID-2019”, “severe acute respiratory syndrome coronavirus 2”, “vaccination”, “bivalent vaccine”, and “updated vaccine“, with Boolean operators and MeSH terms. In order to ensure a comprehensive search and identify the maximum number of potential articles, there were no constraints on language, the year of publication, or participant characteristics. Two authors (N.C. Chiu and C.Y. Lin) performed the literature search independently, and disagreements were resolved through a discussion with the third author (H. Chi).

### 2.2. Study Screening, Data Extraction, Systematic Review, and Meta-Analyses

The inclusion criteria for enrollment were randomized controlled trials or cohort studies investigating bivalent COVID-19 vaccination in humans with all types of platforms. The exclusion criteria were as follows: duplicate publications, irrelevant articles, editorials without clinical data, simple case reports, animal studies, and review articles. The primary outcomes were the immunogenicity or clinical effectiveness of COVID-19 vaccination against moderate-to-severe COVID-19 infection, including hospitalizations, emergency department visits, and mortality. The secondary outcomes were reactogenicity or adverse events. We extracted the following data from the selected studies: the name of the first author, the study country, study type, study period, participant population, participant age/gender, booster vaccine type, control group, immunogenicity profiles, clinical outcomes, safety profiles, predominant viral mutants if available, and the conclusion of the author(s). For quality assessments, we used the Revised Cochrane risk-of-bias tool for randomized trials (RoB 2) for randomized controlled trials and the Newcastle–Ottawa Scale (NOS) for observational cohort studies [20,21]. Two authors assessed quality independently based on selection, ascertainment, causality, and reporting (S.Y. Chen and C.Y. Lin). If a disagreement occurred, a consensus was reached through a discussion with the third author (N.C. Chiu).

BA.4 and BA.5 shared the same spike protein structure, and an updated vaccine comprising an ancestral strain and the BA.4/BA.5 strains was developed to protect against both an ancestral strain and the BA.4/BA.5 strains. In order to reduce redundancy, we used BA.5 to represent BA.4/BA.5, and a BA.5-containing BV indicated an updated vaccine comprising an ancestral strain and the BA.4/BA.5 strains. Furthermore, we assumed the geometric titer (GMT) between different studies was comparable, and a further meta-analysis was conducted to evaluate the immunogenicity of the BV and the original monovalent vaccine (MV). We also contacted authors for original data, if available.

### 2.3. Statistical Analyses

We used a random-effects regression model for the meta-analyses, assuming that the true effect size was not the same. The τ^2^ statistic was used to investigate the heterogeneity of the enrolled studies. Comparison-adjusted funnel plots, contour-enhanced funnel plots, and Egger’s tests were used to examine potential publication bias. A *p*-value less than 0.05 was considered statistically significant. We used MedCalc, version 18 (MedCalc software, Ostend, Belgium) v18 and R software version 4.2.2 with RStudio (R Foundation for Statistical Computing, Vienna, Austria) for the statistical analyses.

## 3. Results

### 3.1. A Flowchart of the Systematic Review

**Figure 1:**
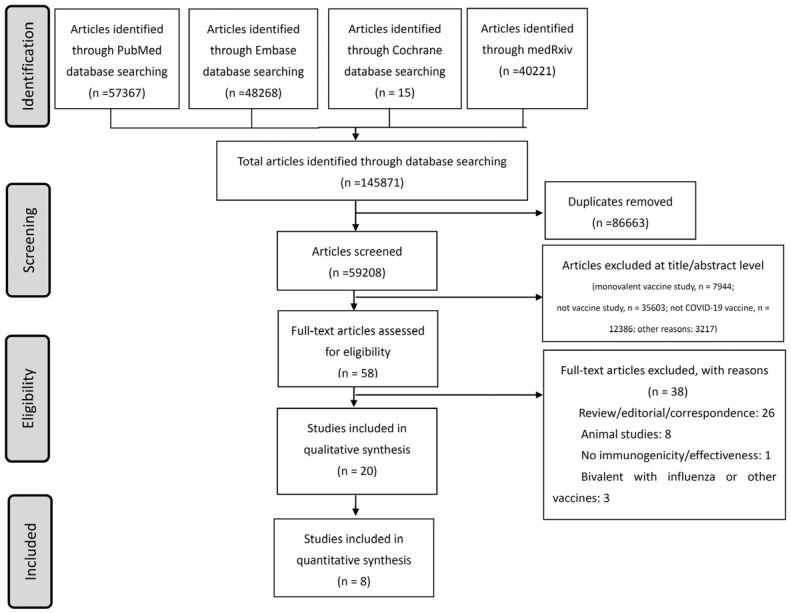
Flowchart of the literature search and the enrolled studies.

As of March 2023, 20 studies were identified for the systematic review (Figure 1) [15,16,22,23,24,25,26,27,28,29,30,31,32,33,34,35,36,37,38,39]. A total of nine studies investigated the effectiveness of a BA.5-containing BV, five studies explored a BA.1-containing BV, and two studies examined a BA.1- or BA.5-containing BV (Table 1). Immunogenicity was described in 13 studies, and clinical effectiveness against COVID-19-related hospitalizations, emergency department visits, or mortalities from observational cohort studies or databases was reported in 7 studies. Most studies were conducted in the United States, and others were conducted in China, Europe, and Israel.

### 3.2. Comparison of Immunogenicity

**Figure 2:**
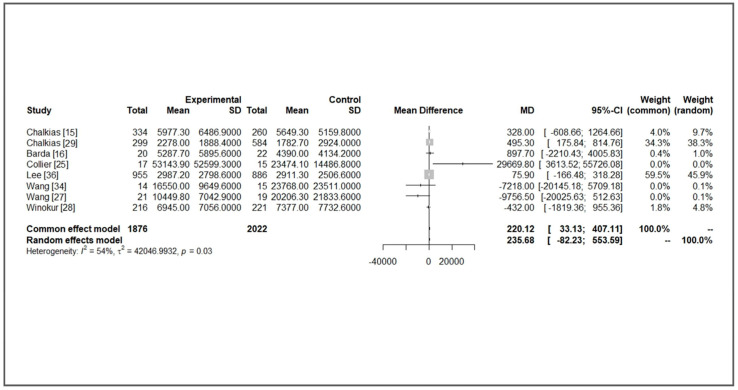
Forest plot of the geometric titer against ancestral strains of bivalent and monovalent vaccines [15,16,25,27,28,29,34,36].

**Figure 3:**
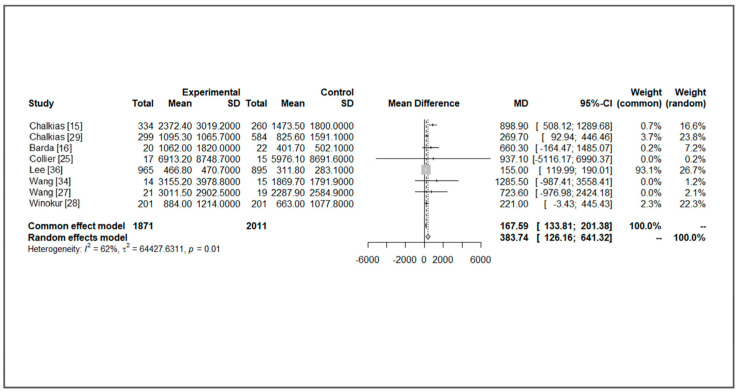
Forest plot of the geometric titer against new additional variants of bivalent and monovalent vaccines [15,16,25,27,28,29,34,36].

**Figure 4:**
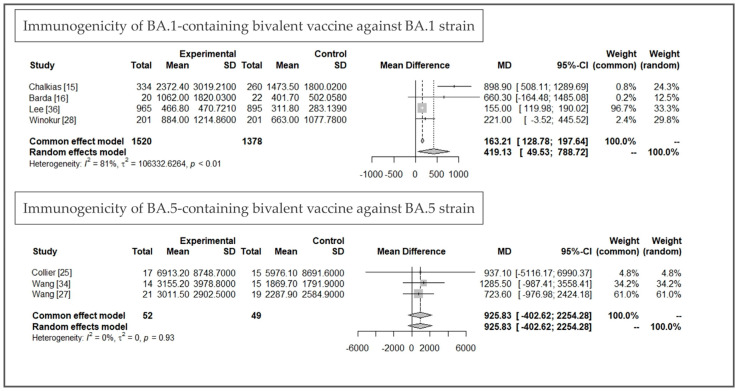
Subgroup analysis of immunogenicity induced by BA.1-containing and BA.5-containing bivalent vaccines [15,16,25,27,28,34,36].

A substantial increase in immunogenicity was observed after all kinds of boosters, including MVs and BVs. Compared with BA.1 or BA.5, the increase in immunogenicity against an ancestral strain was more evident in all enrolled studies. For specific newly additional bivalent strains, most studies showed higher GMT levels in the BV group, but the increased differences were not significant (a GMT ratio from 1.3 to 2.5). For studies with detailed data available, we assumed the immunogenicity was comparable between different studies because the immunological findings were similar. We performed a meta-analysis to investigate the immunogenicity of MV and BV boosters. Vaccine-induced immunogenicity against ancestral strain was similar with moderate heterogeneity between MV and BV groups (mean difference (MD) of GMT: 235.68, 95% confidence interval (CI): −82.83~553.59, *I*^2^: 54%, *p* = 0.03, Figure 2). For immunogenicity against additional variants (Beta, BA.1, or BA.5), BVs had higher GMT titers (MD of GMT: 383.75, 95% CI: 126.16~641.33, *I*^2^: 62%, *p* = 0.01, Figure 3). In subgroup analysis comparing BA.1-containing BVs, a significantly higher GMT was observed in a BA.1-containing BV (MD of GMT: 419.13, 95% CI: 49.53~788.72, *I*^2^: 81%, *p* < 0.01, Figure 4). This benefit was not significant when we compared it with immunogenicity in studies against the BA.5 strain (MD of GMT: 925.93, 95% CI: −402.62~2254.28, *I*^2^: 0%, *p* = 0.93, Figure 4). Further funnel plots showed some asymmetry of the enrolled studies (Supplementary Figures S1 and S2). In brief, BVs showed equal immunogenicity values against the ancestral strain and superior immunogenicity against new additional strains, but the increase was not significant in BA.5-containing BVs. The increase was significant for BA.1-containing BVs (MD 419.13, 95% CI: 49.53~788.72). We summarize the major findings of immunogenicity in Table 2.

### 3.3. Comparison of Clinical Effectiveness

Six observational cohort studies explored the clinical effectiveness of BVs. The estimated vaccine effectiveness against symptomatic infection was in the range of 14~43% (95% CIs from 3 to 24 and from 39 to 46%, respectively). The better protection of BVs against hospitalization was observed (48~73%, with 95% CIs from 30 to 62 and from 52 to 85%, respectively). Most studies reported a 1-month follow-up period; therefore, long-term effectiveness remains unclear.

### 3.4. Comparison of Safety

The reactogenicity was similar between MVs and BVs, and there was no safety concern of the BV in any study. A higher incidence of adverse events was observed in the BA.1-containing BA group in Winokur’s study (from 8.5 to 10.4% vs. from 3.6 to 6.6%), but the adverse events were mild and tolerable [28].

## 4. Discussion

Our systematic review showed robust immunogenicity after an MV or BV booster. From the viewpoint of immunogenicity, the BVs were equal to MVs in eliciting antibodies against both ancestral and new additional variant strains. An additional clinical effectiveness against hospitalization or emergency department visits of approximately 33–50% was observed in people with a BV booster. There was no safety concern in the published studies. Although the beneficial difference of the BV was not vast, our study demonstrated some evidence of the utility of a BV booster during the Omicron era.

Although the BV is not inferior to the MV, it is surprising that the benefits of the BA.5-containing BV against BA.5 strain are not statistically significant and are lower than our expectations. Approximately 33–50% additional protection is elicited by the BV, and the overall clinical effectiveness against new COVID-19 variants is approximately 50–60% [40,41]. In an observational matched cohort study in the Republic of Korea, an additional 12.2% (95% CI: 6.5 to 17.7%) protection against COVID-19 infection was observed in the recipients of a BV [40]. The clinical effectiveness is unsatisfactory and inadequate to protect vaccine recipients from COVID-19 infection. The effects of “imprinting”, or original antigenic sin, may be attributable to unsatisfactory protection [14,42]. Imprinting from initial antigen exposure may alter subsequent immunological responses following a vaccine booster [43,44,45]. Limited immunological breadth following vaccination with an updated BV may occur in people with a previous infection or vaccination. During the study period, it was the third year of the COVID-19 pandemic, and “naïve” people without natural infection or vaccination were rare. Therefore, the human immune system elicits more robust immunity against the initial strain following a booster with an MV or BV. This “first love phenomenon” may explain why the induced immunogenicity against BA.5 is not promising in people who receive a BA.5-containing booster. Our study also demonstrates much higher levels of immunogenicity against ancestral strains than new additional variant strains across enrolled studies. However, the additional benefits of the BV are not promising, and continuous surveillance is warranted. The human immune system is delicate and complicated, and the entire immune mechanism of SARS-CoV-2 infection and vaccination is not fully understood. Although the antibody levels are not significantly different between MV and BA.5-containing BV groups, clinical effectiveness against COVID-19-related hospitalization and mortality was observed in the BV group. Further studies are warranted to elucidate the underpinning mechanisms, the benefits of BV boosters, and the optimal design of future vaccines.

The success of the COVID-19 vaccine against the Delta variant is an important milestone during this long-running battle [7,46,47,48]. However, the rapidly mutating variant results in immune escape and breakthrough infections in vaccinees. Several strategies have been adopted to restore immunity and protection, including the addition of boosters, heterologous administration, dosage adjustment, and interval adjustments. The development of a BV may reduce bottlenecks, but the coverage rate of the BV vaccination among eligible residents is low. As of 31 December 2022, 27.1% of eligible adults had received a BV booster, and 18.5% of adolescents had received a BV booster in the United States [49]. Among the adults and adolescents with parents who were open to receiving a BV booster, 16.9% and 11.8% had concerns about vaccine safety [49]. Therefore, the BV booster coverage was not high, and safety concerns remained an important issue. Our systematic review shows no safety concerns in BV booster recipients and is consistent with the observational cohort study in children and adolescents [50,51]. Furthermore, multiple reasons for not receiving a BV have been identified, including a lack of awareness of eligibility for vaccination, vaccine availability, and perceived immunity against infection [52,53]. Our study demonstrates the equal immunogenicity of the BV to the MV without safety concerns. Approximately 33% additional protection with respect to clinical effectiveness and immunogenicity is provided by the BV booster, with moderate heterogeneity. A joint approach with shared decision making regarding receiving a booster or not is recommended. If receiving a booster is preferred, a BV booster is preferred based on the present study.

Vaccination is considered a game-changer in the ongoing battle against COVID-19. However, the decline in vaccine-induced protection poses a significant challenge, necessitating booster vaccinations to maintain sufficient immunity. A previous systematic review and meta-analysis indicated that individuals who received a booster shot exhibited stronger protection against Omicron compared to those who had only received the primary series, with vaccine effectiveness rates of 53.1% (95% CI: 48.0–57.8%) versus 28.6% (95% CI: 18.5–37.4) against infection [13]. Additionally, for vaccine effectiveness against severe cases, individuals who received a booster also enjoyed greater protection (87.3% versus 57.3%, 95% CI: 75.5–93.4% and 48.5–64.7%, respectively). These findings underscore the critical role of booster vaccinations [8]. Nevertheless, the vaccine effectiveness of bivalent vaccinations remains largely uncharted territory, and our systematic review represents the first comprehensive examination demonstrating the advantages of BVs.

The virus continues to evolve; thus, persistent surveillance and monitoring are crucial to detect new mutants early. Mutants of XBB sub-lineages became predominant in many countries after the spring of 2023 [54,55]. The ability of XBB to transmit is increasing, and the severity after infection is similar. Protection due to vaccination is challenged by new variants. The additional protection of BV against severe COVID-19 varied from 14.3% to 45.6% (95% CI: 1.6–25.3 and 1.6–69.9, respectively) for people with an XBB infection [56,57]. Vaccination with an updated BV is preferred during the XBB era until a new vaccine against the XBB sub-lineage is available. However, viral mutation continues, and continuous surveillance is required to investigate vaccination protection.

Our review demonstrates an early estimate of the immunogenicity and clinical effectiveness of BV boosters with real-world evidence. However, our study was subject to some limitations. First, most studies had a short follow-up period, and the duration of BV booster protection remains unclear. It is reasonable that a waning of the mRNA vaccine occurs, but further studies are required to identify the duration of protection. Furthermore, most studies were observational studies with a limited availability of randomized controlled trials. Consequently, calculating a precise odds ratio can be challenging. Second, there are two kinds of BA.5-containing BV, and a detailed comparison between these two vaccines has not been performed. Third, the intervals between the primary series, the number of previous vaccinations, the natural infection status, the immune status, and age groups are inconsistent across studies, and bias may exist. More than 670 million residents have a history of COVID-19, and naïve people without COVID-19 or vaccination against COVID-19 were difficult to identify. People with a history of previous infection or vaccination may have impacted the subsequent effectiveness of vaccination. Fourth, the emergence of mutants, such as the BA.2.75, XBB, BQ.1, and EG.5 subvariants, is ongoing; immunogenicity and clinical effectiveness may be challenged by new variants [58]. Finally, the BV has been authorized to be administered as a booster dose. The administration of the BV as a primary series may mitigate the effects of imprinting and elicit higher specific immune responses of additional vaccine strains. However, the safety, immunogenicity, and true effects of the BV as a primary immunization have not been investigated.

## 5. Conclusions

In conclusion, our systematic review showed that BVs have equal immunogenicity against the ancestral strain. Compared with the MV booster, the BV booster demonstrated approximately 33–50% additional immunogenicity against additional variants and clinical effectiveness. There were no safety concerns. The present study provides preliminary evidence of the clinical effectiveness of the BV booster and contributes to the informed preferences of physicians and the public to receive a booster vaccine. Shared decision making is recommended for receiving a booster against COVID-19; if a booster is preferred, the BV booster is suggested. Further studies are required to investigate the durability of the BV booster and the effectiveness of the BV against new variants. Continuous surveillance is warranted to detect viral mutations and adjust vaccination policy.

## Figures and Tables

**Table 1:** Demographic characteristics of the enrolled studies investigating the immunogenicity or effectiveness of updated bivalent COVID-19 vaccines.

Study	Country	Study Design	Study Period	Bivalent	Comparator	Population	Outcomes	Conclusion	Safety Concern
Protein-Based Inactivated Vaccine									
He [31]	China	Randomized controlled trial	2022/08~2022/09	V-01D-351 (Beta + Delta) BV-01-B5 (ancestral + Omicron)	Inactivated monovalent vaccine	56 participants	Safety and immunogenicity	The bivalent boosters induced robust antibody responses against multiple Omicron sublineages (GMR: 8.5~31.3).	No
Bivalent BA.1-containing mRNA Vaccine									
Barda N [16]	Israel	Phase 3 trial	ND	Ancestral + BA.1	Monovalent	122 elders	Immunogenicity	BA.1-adapted mRNA vaccines led to a stronger neutralizing antibody response against the Omicron BA.1 subvariant (GMR: 2.07 [0.93–4.58]).	No
Chalkias S, NEJM [15]	USA	Phase 2/3 study	2022/02–2022/03	Ancestral + BA.1	Monovalent vaccination	812 adults	Safety, reactogenicity, and immunogenicity	High GMT titers of bivalent vaccinations (against BA.1: 2372.4 [2070.6 to 2718.2] vs. 1473.5 [1270.8 to 1708.4]; against BA.4/5: 727.4 [632.8 to 836.1] vs. 492.1 [431.1 to 561.9]).	No
Huiberts AJ [30]	Netherlands	2022/09~2022/12	Prospective cohort study (VASCO)	Ancestral + BA.1	Monovalent	32,542 adults	Infection incidence	The overall bivalent vaccine effectiveness was 31% [18–42] (18–59 years) and 14% [3–24] (60–85 years).	No
Lee IT [36]	UK	Phase 2/3 trial	2022/04–2022/06	Ancestral + BA.1	Monovalent vaccination	1871 adults	Safety, reactogenicity, immunogenicity	High GMT titers of bivalent vaccinations (against BA.1: GMR 1.53 [1.41–1.67]; against ancestral: GMR 1.05 [0.96–1.15]).	No
Winokur P [28]	Several countries	Phase 3 trial	2022/03~2022/04	Ancestral + BA.1	Monovalent	1846 adults older than 55 years	Immunogenicity	BA.1-adapted vaccines induced substantial neutralizing responses against Omicron BA.1 strains (GMR: 1.56 [1.17–2.08] and 1.97 [1.45–2.68]) and ancestral strain, and, to a lesser extent, neutralized the BA.4, BA.5, and BA.2.75 strains.	No
Bivalent BA.5-containing mRNA Vaccine									
Anft M [33]	Germany	Cohort study	ND	Ancestral + BA.5	Nil	35 hemodialysis patients	Immunogenicity	Strong immune responses after booster.	No
Collier AY [25]	USA	Cohort study	ND	Ancestral + BA.5	Monovalent	33 adults	Cellular and humoral immunity	Both the monovalent and bivalent mRNA boosters markedly increased antibody responses.	No
Huth L [26]	Germany	Cohort study	ND	Ancestral + BA.5	Nil	55 hemodialysis patients	Immunogenicity	Significant increase after booster (7.3× increase in anti-spike IgG concentrations in those had no previous omicron infection).	No
Lin DY [39]	USA	Observational cohort study	2022/09~2022/12	Ancestral + BA.5	Monovalent vaccine	292,659 + 1,070,136 adults	COVID-19 hospitalizations, deaths	Hospitalizations: Monovalent: 25.2% [−0.2 to 44.2] Bivalent: 58.7% [43.7 to 69.8] Severe infection: Monovalent: 24.9% [1.4 to 42.8] Bivalent: 61.8% [48.2 to 71.8]	No
Link-Gelles [22]	USA	Observational cohort study (ICATT)	ND	Ancestral + BA.4/BA.5	Unvaccinated; monovalent vaccinations	360,626 adults	Symptomatic infection	Absolute vaccine effectiveness: 22 [15–29]~43 [39–46]%	No
Surie D [23]	USA	Observational study (IVY)	2022/09–2022/11	Ancestral + BA.4/BA.5	Unvaccinated; monovalent vaccinations	798 immunocompetent adults aged > 65 years	Hospitalizations	Unvaccinated: 84% [64–93] MV: 73% [52–85]	No
Tenforde MW [24]	USA	Observational study (VISION Network)	2022/09~2022/11	Ancestral + BA.4/BA.5	Unvaccinated; monovalent vaccinations	78,170 immunocompetent adults	Emergency visits and hospitalizations	Vaccine effectiveness against ED visits: Unvaccinated: 56% [49–62] MV: 50% [43–57] Hospitalizations: Unvaccinated:59% [44–70] MV: 48% [30–62]	No
Wang Q, NEJM [27]	USA	Cohort study	ND	Ancestral + BA.5	Monovalent vaccine; breakthrough infection	41 adults	Immunogenicity	Boosting with the bivalent mRNA vaccines is not evidently better than boosting with the original monovalent vaccine (neutralization antibody 1649 vs. 1366, *p* = 0.57).	No
Wang Q, LID [34]	USA	Cohort study	2022/09~2022/10	Ancestral + BA.5	Unvaccinated, monovalent vaccine, breakthrough infection	74 adults	Immunogenicity	The bivalent booster did not elicit a discernibly superior virus-neutralizing peak antibody response (neutralizing antibody 835 vs. 509, *p* = 0.22).	No
Bivalent BA.1- or BA.5-containing mRNA Vaccine									
Canaday DH [37]	USA	Cohort study	2022/09~2022/11	Ancestral + BA.1 or BA.5	Monovalent vaccination	261 participants (nursing homes)	Immunogenicity	The bivalent booster substantially elevated neutralizing antibody titers against the Wuhan, BA.1, and BA.4/BA.5 strains (BA.4/5 before vs. after = 160 vs. 1964, *p* < 0.01).	No
Johnson AG [38]	USA	Retrospective surveillance data	2021/03~2022/12	Ancestral + BA.1 or BA.5	Unvaccinated or monovalent	21,296,326 COVID-19 cases	COVID-19 incidence and mortality	The bivalent booster recipients in 24 US jurisdictions had slightly higher protection against infection and significantly higher protection against death (for people aged 65–79, a risk ratio of 23.7 [12.6–44.7] was observed in unvaccinated people).	No
Bivalent mRNA Vaccine with other Components									
Chalkias S, Nat Med [29]	USA	Phase 2/3 study	2021/05~2021/07	Ancestral + Beta	Monovalent vaccination	895 + 584 + 171 adults	Safety, reactogenicity, immunogenicity	Higher immunogenicity of bivalent vaccinations (GMR: 1.23 [1.01–1.50]~2.74 [2.22–3.40]).	No
Dayan GH [35]	8 countries	Phase 3 trial	2021/10~2022/02	Ancestral + Beta	Placebo	12,924 adults	Symptomatic infection	A vaccine efficacy 64.7% [46.6 to 77.2].	No
Hannawi S [32]	China	Phase 1/2	2022/01~2022/04	Alpha + Beta	Placebo	234 adults	Reactogenicity, immunogenicity	The SCTV01C booster was safe with reactogenicity profiles.	No

Abbreviations; [ ], 95% confidence interval; BV, bivalent vaccine; COVID-19, coronavirus disease 2019; ED, emergency department; GMR, geometric mean ratio; GMT, geometric mean titer; ICATT, increasing community access to testing; IVY, investigating respiratory viruses in the Acute Ill Network; MV, monovalent vaccine; ND, not described; VISION network, virtual network to investigate risk of COVID-19-associated outcomes and COVID-19 vaccine effectiveness using integrated medical and public health records.

**Table 2:** Mean geometric mean titers of bivalent vaccines against ancestral or new additional variants in some studies.

	Monovalent Vaccines vs. Bivalent Vaccines (BA.1 or BA.5)				Monovalent Vaccines vs. Bivalent Vaccines (BA.1)				Monovalent Vaccines vs. Bivalent Vaccines (BA.5)			
	Against New Variant		Against Ancestral Strain		Against BA.1		Against Ancestral Strain		Against BA.5		Against Ancestral Strain	
Vaccine	BV	MV	BV	MV	BV	MV	BV	MV	BV	MV	BV	MV
GMT	1065.9	719.8	4526	3911.4	948.5	583.7	4232.8	4157.8	4325.7	3288.9	26,049.9	22,297
MD	383.75		235.68		419.13		81.68		925.83		1416.82	
95% CI	126.16	641.33	−82.23	553.59	49.53	788.72	−149.04	312.4	−402.62	2254.28	−20,408.9	23,242.53

Abbreviations: BV, bivalent vaccine; CI, confidence interval; GMT, geometric mean titer; MD, mean difference; MV, monovalent vaccine.

## Data Availability

The datasets used for analysis in the present study are available from the corresponding author upon reasonable request.

## Supplementary Materials

The following supporting information can be downloaded at https://www.mdpi.com/article/10.3390/life13102094/s1. Figure S1: The funnel plot of included studies investigating immunogenicity against ancestral strain. Figure S2: The funnel plot of included studies investigating immunogenicity against additional variants.
