# Supplementary figures and images for: The Effectiveness of Bivalent COVID-19 Vaccination: A Preliminary Report
Source: Life (Basel). 2023 Oct 21;13(10):2094. doi: 10.3390/life13102094 (PMC10608313; doi:10.3390/life13102094)

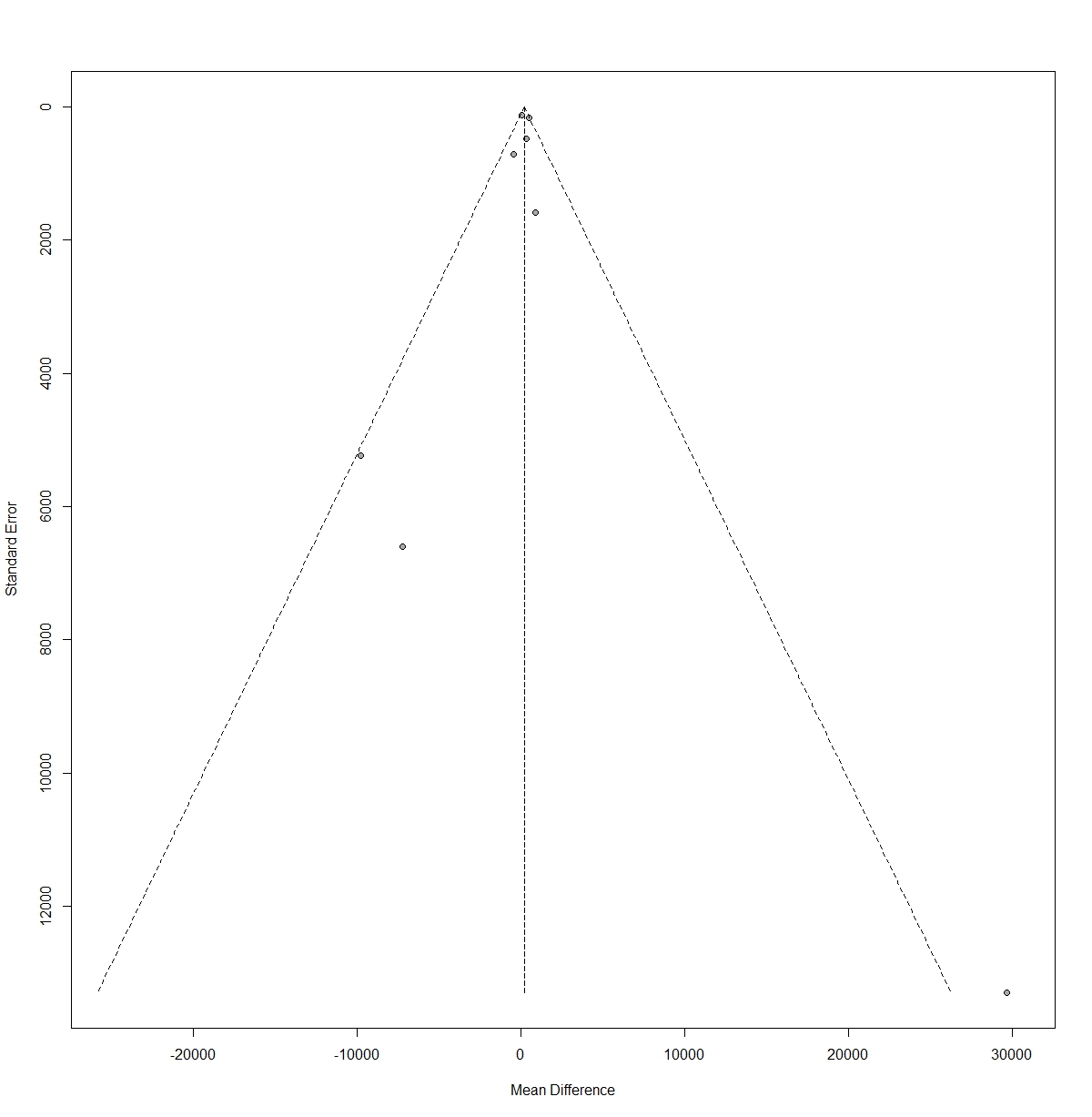

Supplement: Supplementary file 1 [file life-13-02094-s001.zip › Supplementary Figure S1_Ancestral_funnel.jpeg]

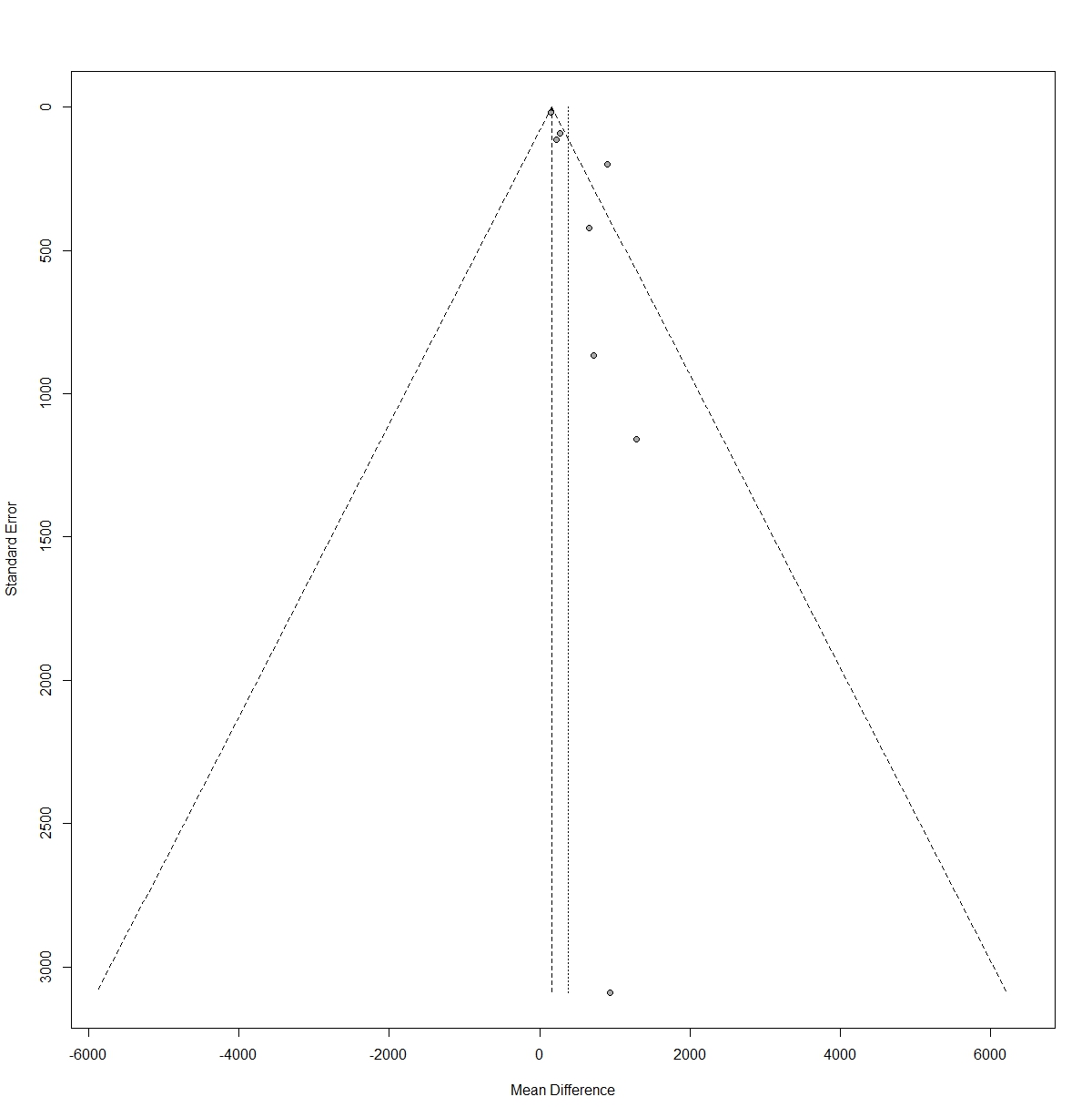

Supplement: Supplementary file 1 [file life-13-02094-s001.zip › Supplementary Figure S2_Bivalent_funnel.jpeg]
